# Mechanistically based mapping of human cardiac fibrillation

**DOI:** 10.1113/JP270513

**Published:** 2016-01-19

**Authors:** Sanjiv M. Narayan, Junaid A. B. Zaman

**Affiliations:** ^1^ Stanford UniversityPalo AltoCAUSA; ^2^ Imperial College LondonLondonUK

## Abstract

The mechanisms underpinning human cardiac fibrillation remain elusive. In his 1913 paper ‘On dynamic equilibrium in the heart’, Mines proposed that an activation wave front could propagate repeatedly in a circle, initiated by a stimulus in the vulnerable period. While the dynamics of activation and recovery are central to cardiac fibrillation, these physiological data are rarely used in clinical mapping. Fibrillation is a rapid irregular rhythm with spatiotemporal disorder resulting from two fundamental mechanisms – sources in preferred cardiac regions or spatially diffuse self‐sustaining activity, i.e. with no preferred source. On close inspection, however, this debate may also reflect mapping technique. Fibrillation is initiated from triggers by regional dispersion in repolarization, slow conduction and wavebreak, then sustained by non‐uniform interactions of these mechanisms. Notably, optical mapping of action potentials in atrial fibrillation (AF) show spiral wave sources (rotors) in nearly all studies including humans, while most traditional electrogram analyses of AF do not. Techniques may diverge in fibrillation because electrograms summate non‐coherent waves within an undefined field whereas optical maps define waves with a visually defined field. Also fibrillation operates at the limits of activation and recovery, which are well represented by action potentials while fibrillatory electrograms poorly represent repolarization. We conclude by suggesting areas for study that may be used, until such time as optical mapping is clinically feasible, to improve mechanistic understanding and therapy of human cardiac fibrillation.

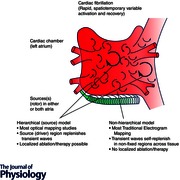

**Abbreviations**
APD: action potential duration
AF: atrial fibrillation
DI: diastolic interval
FIRM: focal impulse and rotor mapping
MAP: monophasic action potential
VF: ventricular fibrillation

## Introduction

Cardiac fibrillation encompasses the most complex currently appreciated electrical disorders of the heart, whose rapidly changing spatiotemporal patterns challenge our mechanistic understanding. In many ways, fibrillation represents a ‘final frontier’ in arrhythmia medicine.

Atrial fibrillation (AF) is the most common sustained arrhythmia in the world, affecting over 30 million individuals worldwide (Chugh *et al*. 2014), and is a major cause of hospitalizations, stroke and death. Therapy includes drugs to modulate cellular or membrane electrical function, or ablation to eliminate pro‐arrhythmic tissue, yet both remain suboptimal due to uncertain mechanistic targets and side‐effects on bystander myocardium (January *et al*. 2014).

Ventricular fibrillation (VF) is a major cause of sudden cardiac arrest, which afflicts over 300,000 individuals per year in the USA (Chugh *et al*. 2004) and a similar number in Europe. Pharmacological therapy is limited, and even drugs with sound scientific rationale can increase mortality, highlighting dangerous gaps in our understanding (The Cardiac Arrhythmia Suppression Trial (CAST) Investigators, 1989). Non‐pharmacological therapies are also suboptimal and primarily comprise ablation of potentially large areas of viable tissue with modest success rates or cessation of an episode of fibrillation by high‐energy electric field‐shock that does not prevent future episodes.

Improved mechanistic definition is essential to improve therapy. A central debate in human atrial or ventricular fibrillation is whether disordered activity is driven by organized processes (hierarchical model) or is self‐sustaining (non‐hierarchical model). Fibrillatory wavelets have a stochastic probability of terminating upon encountering a boundary, and must therefore be replenished to sustain fibrillation (Chen *et al*. 2000 *a*). In the first model wavelets are replenished by spatially localized mechanisms, at preferred ‘source’ regions, whereas in the second model wavelets self‐replenish, indicating no preferred regional source(s). This dichotomy is of fundamental importance because it dramatically influences mechanistic thinking at the genetic and cellular levels, structure–function relationships, imaging and therapy.

It is striking not only how little overlap exists between camps, but that this dichotomy may also be divided by mapping technique. Focusing on AF, whilst optical mapping studies of regional activation and recovery (action potentials) mostly show stable source regions that drive disordered activity, most studies that map surrogates for local activation (bipolar or unipolar electrograms) do not, as summarized in Table 1.

**Table 1:** **Differences in atrial fibrillation mechanisms by mapping modality**

			Surface (field	Spatial		Quoted mechanism
Study	Species	Chamber	of view)	resolution	Signals	in original study
**Atrial – electrogram**						
Cox *et al*. (1991)	Human	RA and LA	Epicardial (narrow field)	6 mm	EGM (bipolar)	‘Single reentrant circuit’ in RA, LA ‘reentry could not be detected’.
Schuessler *et al*. (1992)	Dogs	RA	Epicardial (narrow field)	5 mm	EGM (bipolar)	‘Multiple reentrant circuits stabilized into a single small circuit by ACh’.
Konings *et al*. (1994)	Human	RA	Epicardial (narrow field)	2.25 mm	EGM (unipolar)	(1) ‘Reentry (random/leading circle)’. (2) ‘Epicardial breakthrough’.
Holm *et al*. (1997)	Human	RA	Epicardial (narrow field)	3 mm	EGM (bipolar)	(1) ‘Organized (non‐random) re‐entry’. (2) ‘Focal’.
Schilling *et al*. (2000)	Human	RA	Endocardial (wide field)	N/A	Virtual EGM from non‐contact balloon	(1) ‘Single reentry’. (2) ‘Multiple wavefronts’.
Wu *et al*. (2002)	Human	RA and LA	Epicardial (narrow field)	3 mm	EGM (bipolar)	(1) ‘Large wavefront in RA’. (2) ‘Rapid repetitive activities in LA’.
Sahadevan *et al*. (2004)	Human	RA and LA	Epicardial (series of plaques)	1.2 mm	EGM (bipolar)	(1) ‘Driver with fibrillatory conduction’. (2) ‘No observable pattern’.
Houben *et al*. (2004)	Human	RA	Epicardial (narrow field)	2.25 mm	EGM (unipolar)	(1) ‘Multiple wavefronts’. (2) ‘A stable reentrant circuit was not seen’.
Allessie *et al*. (2010)	Human	RA	Epicardial (narrow field)	2.25 mm	EGM (unipolar)	(1) ‘We failed to find any rotors or foci that could explain the persistence of AF’. (2) ‘Longitudinal dissociation’.
de Groot *et al*. (2010)	Human	RA	Epicardial (narrow field)	2.25 mm	EGM (unipolar)	(1) ‘Complete reentrant circuits in the epicardial plane were extremely rare’. (2) ‘Epicardial breakthrough’.
Narayan *et al*. (2012 *d*)	Human	LA and RA	Endocardial (wide field)	4 mm	Computational physiological filtering of EGMs (FIRM)	(1) ‘Localized rotors’*. (2) ‘Focal impulses’*.
Eckstein *et al*. (2013)	Goat	LA	Endocardial and epicardial (narrow field)	1.6 mm	EGM (unipolar)	(1) ‘Full 360° rotation was found in < 1% of all 3944 waves’. (2) ‘Endo‐epi dissociation’.
Haissaguerre *et al*. (2013)	Human	LA and RA	Epicardial (wide)	5–10 mm	Virtual EGM from body surface	(1) ‘Unstable rotors’.
Lee *et al*. (2013)	Canine	RA and LA	Epicardial (narrow field)	1.2 mm	EGM (bipolar)	(1) ‘Multiple foci’. (2) ‘No random reentry’. (3) ‘Ordered reentry infrequent’.
Miller *et al*. (2014)	Human	LA and RA	Endocardial (wide)	4 mm	Computational physiological filtering of EGMs (FIRM)	(1) ‘Patient specific rotor and focal sources’*.
Lee *et al*. (2014)	Human	LA	Epicardial (narrow field)	2.5 mm	EGM (bipolar)	(1) ‘Multiple unstable wavefronts’. (2) ‘Disorganized activity’. (3) ‘Transient rotational circuits’.
Walters *et al*. (2015)	Human	LA	Epicardial (narrow field)	2.5 mm	EGM (bipolar)	(1) ‘Transient rotors and focal activations’.
**Atrial – optical**						
Gray *et al*. (1996)	Sheep	RA	Epicardial and transmural	0.5 mm	Optical action potentials	(1) ‘Incomplete reentry’. (2) ‘Epicardial breakthrough’.
Skanes *et al*. (1998)	Sheep	RA and LA	Epicardial	0.12 mm	Optical action potentials	(1) ‘Stationary rotors’*. (2) ‘Periodic breakthroughs’.
Berenfeld *et al*. (2000)	Sheep	RA and LA	Epicardial	0.5 mm	Optical action potentials	‘Microreentrant sources in the LA with fibrillatory conduction’*.
Sarmast *et al*. (2003)	Sheep	RA and LA	Epicardial	0.5 mm	Optical action potentials	‘LA and RA rotors’*.
Po *et al*. (2005)	Canine	LA/PV	Endocardial	0.11 mm	Optical action potentials	‘Single stationary PV reentrant circuits conforming with rotor hypothesis’*.
Chou *et al*. (2011)	Canine	LA/PV	Epicardial	0.24 mm	Optical action potentials	‘Epicardial ablation of LA rotor anchoring sites suppresses AF’*.
Gutbrod *et al*. (2015)	Sheep	LA	Epicardial and transmural	0.5 mm	Optical action potentials	(1) ‘Short lived meandering rotors’. (2) ‘Transmural discordance’.
Hansen *et al*. (2015)	Human	RA	Epicardial and transmural	0.33 mm	Optical action potentials	(1) ‘Single localized micro‐anatomic re‐entry can sustain AF in the human heart ex vivo and support the localized driver hypothesis’*.
Zhao *et al*. (2015)	Human	LA	Epicardial and transmural	0.33 mm	Optical action potentials	(1) ‘Stationary reentrant AF drivers’*.

*Studies showing stable fibrillatory sources. Endo, endocardium; epi, epicardium; LA, left atrium; PV, pulmonary vein; RA, right atrium.

This review sets out to reconcile how mapping techniques may diverge in fibrillation due to the functional information they convey. We first review results from optical mapping (Gray *et al*. 1998), often considered the gold standard (Efimov *et al*. 2004). These studies highlight re‐entry (Spach, 2001; Kléber & Rudy, 2004) that emphasize Mines's seminal work on dynamic equilibrium (Mines, 1913), which defined basic criteria for re‐entrant arrhythmias and introduced the concept of a vulnerable period. The centenary of this work was marked in the *The Journal of Physiology* (Paterson, 2013) and its continued importance is clear from the depth and breadth of articles in this special issue. Second, we discuss how various mapping approaches that are concordant in organized rhythms may diverge in fibrillation, focusing on the commonly mapped and ablated rhythm of AF. Third, given recent optical mapping studies of human cardiac fibrillation, we attempt to reconcile the results of clinical mapping in human AF and VF. We conclude by suggesting fruitful areas for future study.

## Dynamic equilibrium and human cardiac fibrillation

Dynamic and fixed mechanisms are essential ingredients of paroxysmal arrhythmias (Weiss *et al*. 2015). Under certain conditions, a single ectopic beat can initiate supraventricular tachycardia using stable anatomical pathways, yet under different conditions the ectopic beat may remain orphaned. These concepts also apply to AF or VF, in which dynamic triggers such as ectopic beats, short bursts of tachycardia or varying autonomic balance may initiate fibrillation despite relatively unchanging cardiac architecture, surface curvature, fibre angles, fibrosis and other ‘fixed’ substrates (Engelman *et al*. 2010).

Dynamic factors can be considered in the context of Mines's *dynamic equilibrium* of activation and recovery. His original definition describes what we may currently call restitution of these fundamental determinants of reentry: ‘For the beating heart is in a complex dynamic equilibrium. The character of each individual beat depends, *inter alia*, upon the lapse of time between that beat and its predecessors.’

This review uses this concept as a foundation to understand fibrillation – in deference to Mines, but also because this equilibrium is mostly neglected in studies of human cardiac fibrillation. Few clinical studies have defined the rate response (restitution) of repolarization or conduction leading up to or during fibrillation, and fewer still incorporate these concepts dynamically into mapping.

**Figure 1:**
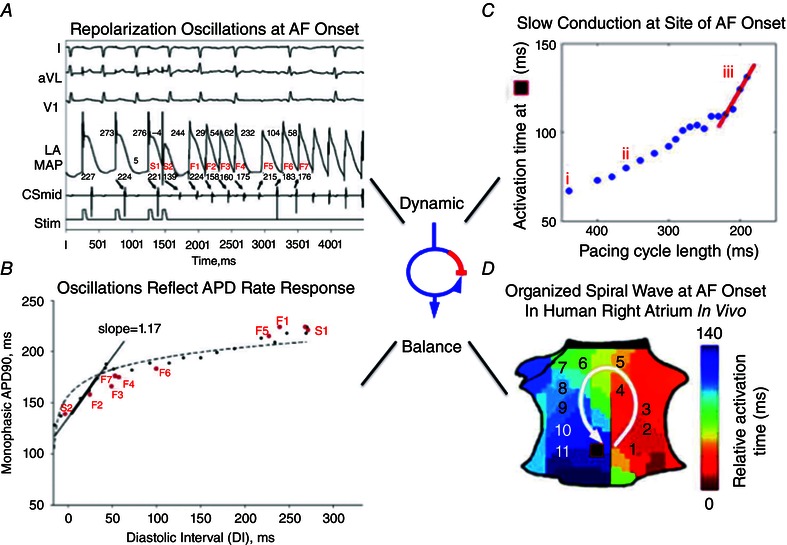
**Dynamic balance between activation and recovery in initiating human atrial fibrillation** *A*, electrograms show ectopic beat (S2) causing AF (varying cycle lengths). *B*, steep APD restitution curve enables S2 to produce repolarization oscillations preceding AF. *C*, conduction restitution curve shows dynamic slowing at site of AF onset just prior to AF onset (iii, red slope). *D*, critical activation delay enables block and formation of a counter‐clockwise spiral initiating AF in right atrium. (From Narayan *et al*. 2008 *b*; Schricker *et al*. 2014.)

The relevance of dynamic equilibrium to clinical fibrillation is shown in Fig. 1, illustrating mechanisms enabling a trigger to initiate human AF. In Fig. 1 *A*, a single ectopic beat initiates human AF during dramatic oscillations of left atrial action potential duration (APD) (Narayan *et al*. 2002, 2008 *b*, 2011 *a*). Figure 1 *B* show that APD oscillations can be explained by rate response of electrical recovery – i.e. restitution curves of APD against diastolic interval (DI, time between successive activations) (Franz *et al*. 1988; Franz, 2003). APD shortens with DI, but in this case its slope of > 1 enabled an early beat (i.e. short DI) to amplify subsequent APD shortening, which amplified DI lengthening for the next beat and so on. Such APD alternans has been shown in animal models (Elzinga *et al*. 1981), and has been shown in patients to enable initiation of AF in proportion to arrhythmic risk (Fig. 1) (Narayan *et al*. 2002, 2008 *b*, 2011 *a*), ventricular tachycardia (Koller *et al*. 2005; Narayan *et al*. 2007) and VF (Karagueuzian *et al*. 1993; Gelzer *et al*. 2008).

Conduction velocity is also rate dependent, and Fig. 1 *C* shows that triggers (in this case, tachycardia) may dramatically slow conduction imminently before AF initiation. Thus, the dynamic equilibrium of APD (repolarization) dispersion and conduction slowing can enable a trigger to produce spiral‐wave re‐entry and initiate human AF (Fig. 1 *D*) (Schricker *et al*. 2014).

This concept can also explain the arrhythmic impact of interventions. For instance, cholinergic stimulation (an AF trigger) shortens APD (Po *et al*. 2005), flattens APD restitution in canine atria (Burashnikov & Antzelevitch, 2005) and slows conduction (Shumaker *et al*. 1993) – particularly if spatially non‐uniform. Mines's concept of dynamic equilibrium therefore provides a useful model for dissecting tractable components of cardiac fibrillation.

## Mapping cardiac fibrillation using dynamics of activation and recovery, and using electrograms

**Figure 2:**
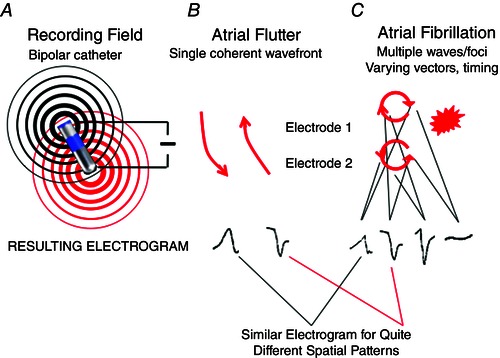
**Quantitative analysis of electrogram morphology** *A*, poles of a catheter (bipolar – close, unipolar – distant) record from distinct mapping fields. *B*, electrograms reflect single wavefront of an organized rhythm (e.g. atrial flutter). *C*, fibrillation, characterized by an uncertain number of wavefronts of uncertain rate, relative timing (phase) and spatial size in undefined recording fields. Summation of these waves may produce variable electrograms from the same spatiotemporal mechanism, or similar electrograms from variable mechanisms. Accordingly, ‘qS’, ‘rR’, or other electrogram rules in fibrillation are not specific for any particular mechanism. The same argument may apply to unipolar electrograms, which summate across wider regions of tissue.

**Figure 3:**
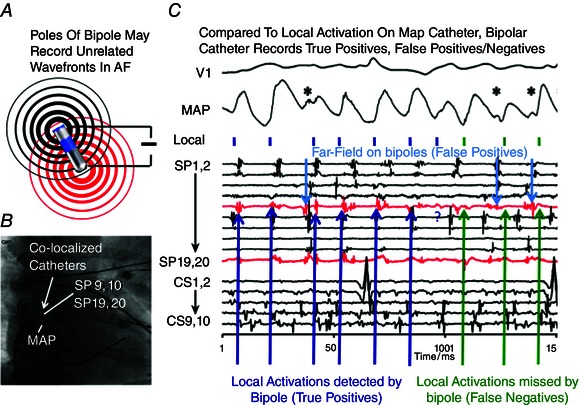
**Limitations of electrogram based activation mapping in AF** *A*, poles of a clinical bipolar electrode may record unrelated wavefronts in AF. *B*, fluoroscopic view of co‐localized MAP, bipolar catheters in human atrium. *C*, MAP in human right atrium indicate local activation (small vertical bars) from far field (asterisks). Notably, bipolar signals (in red) can indicate actual local activation (true positives), show no deflection (false negatives) and show signals that reflect far‐field electrograms (false positives). (Modified from Narayan *et al*. 2011 *b*.)

Fibrillation is a rapid spatiotemporally varying arrhythmia that operates at the limits of activation and recovery. Ideally, therefore, mapping of fibrillation should identify activation and recovery at high temporal resolution and at multiple spatial sites without contamination (cross‐talk) from adjacent sites. Figure 2 summarizes the concept of cross‐talk between an electrode and its reference (Fig. 2 *A*). In spatially coherent rhythms such as flutter where atrial regions activate 1:1 (Fig. 2 *B*), cross‐talk may have limited impact such as altering activation onset time, but as adjacent regions start to reflect unrelated, out‐of‐phase (incoherent) wavefronts in fibrillation (Figs 2 *C* and 3) the impact of cross‐talk is more dramatic and may not only alter activation onset times but even spuriously introduce signals or obscure local activation (Fig. 3).

**Figure 4:**
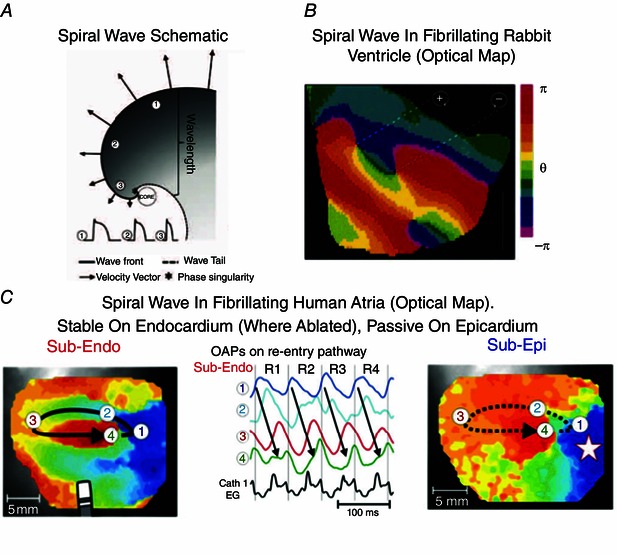
**Spiral wave re‐entry as drivers of cardiac fibrillation** *A*, schematic diagram of spiral wave, showing wavefront curvature as conduction velocity slows towards core (*), where wave front meets wave back. Action potentials from sites 1–3 show varying APD, allowing re‐entry around the unexcited, yet excitable core. (From Pandit & Jalife, 2013.) *B*, first experimental demonstration of spiral waves in rabbit VF. Phase is depicted in colour with spiral wave chirality indicated by + (clockwise) or – (counter‐clockwise). Three phase singularities (PS) are seen. (From Gray *et al*. 1998.) *C*, optical mapping of human atria shows stable micro‐reentrant sources on the endocardium sustaining AF, anchored to endocardial fibre complexity, yet passive, transient activity on the epicardium and elsewhere in the periphery. Optical action potentials (OAPs) at sites 1–4 on the endocardium show activation and recovery over multiple sequential cycles, yet electrograms (Cath 1 EG) vary due to cross‐talk and other factors. The authors concluded that stable endocardial micro‐reentrant sources produce unstable epicardial activations. (From Hansen *et al*. 2015.)

Optical mapping of voltage‐sensitive dyes currently provides the highest spatial resolution of both activation and recovery, with minimal cross‐talk. The approach illuminates the biological field with light to excite voltage‐sensitive dyes, that fluoresce in proportion to membrane potential, i.e. optical action potentials (OAPs) (Fig. 4). Successive images thus map propagation of activation (wavefront) and recovery (waveback). Crucially, optical maps show minimal cross‐talk – activity is represented by visually defined pixels relatively uninfluenced by remote regions. Limitations of optical mapping include the need to immobilize tissue, to minimize absorption of reflected light (e.g. by blood) and toxicity of fluorescent dyes. As a result, optical mapping is not yet clinically feasible, although proof of concept studies in small animals suggest that this may be on the horizon (Lee *et al*. 2012).

In clinical studies, activation and recovery (action potentials) can be measured from an extracellular monophasic action potential (MAP) catheter. Activation and repolarization measurements from MAPs are validated against intracellular recordings (Franz *et al*. 1990), reflect minimal far‐field activity (Knollmann *et al*. 2002), and their dynamics provide insights into human AF (Fig. 1) (Narayan & Franz, 2007; Narayan *et al*. 2011 *b*) or VF (Swartz *et al*. 1993). However, challenges in repeatedly recording MAPs at multiple sites in humans has limited this approach to a small number of experienced centres (Kim *et al*. 2002; Watanabe *et al*. 2002; Narayan & Franz, 2007).

In the clinical setting, for practical reasons, cardiac fibrillation is thus mostly mapped from electrograms – voltage–time series at one electrode relative to an adjacent (bipolar) or remote (unipolar) electrode. Electrogram analyses developed for coherent rhythms provide high temporal resolution that is easily repeated at multiple spatial sites. What is frequently overlooked in fibrillatory electrograms, however, is their dramatic potential for intra‐chamber cross‐talk (Narayan *et al*. 2011 *b*). In Fig. 3 *C*, bipolar AF electrograms include unclear components of local (desired) activity and far field (undesired) activity. Accordingly, AF electrograms may not be sensitive nor specific for local activation on adjacent MAPs – indeed, electrograms may even be absent at times when MAPs show clear local activation due to directionality and/or algebraic cancellation (Fig. 3) (Narayan *et al*. 2011 *b*). Moreover, action potentials encode information about activation and recovery while electrograms focus on activation, with the activation recovery interval measurable only during tightly controlled conditions (Haws & Lux, 1990) that are absent during fibrillation.

**Figure 5:**
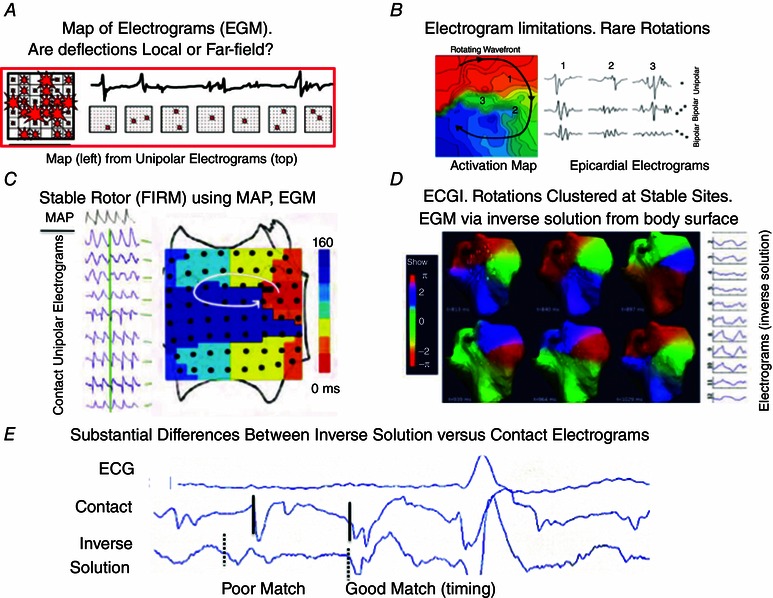
**Human AF maps show differing mechanisms based on mapping technique** *A*, electrograms from epicardial plaque produce complex maps in human AF. Electrograms used for maps (top) illustrate the challenge of assigning local activity with confidence. No rotational wavefronts were seen in > 4000 maps. (From de Groot *et al*. 2010.) *B*, rotor on activation map (early to late) from same group as *A*, which were sensitive to electrogram type and unstable in this study. (From Lau *et al*. 2015.) *C*, focal Impulse and rotor maps (FIRM) showing rotor in human AF, using computational reconstruction of activation and recovery (from physiological MAP and conduction restitution). A stable rotor is identified from phase mapping (here depicted by early meets late activation) that was eliminated by ablation. FIRM‐guided ablation may improve the results of conventional ablation. (From Narayan *et al*. 2012 *c*.) *D*, non‐invasive mapping using the inverse solution show AF sources (progression of phase colours), clustered in stable spatial regions that were treated by localized ablation. Causes for electrical instability at fixed spatial areas may reflect epicardial variability from stable endocardial rotors, technical limitations or other factors. (From Haissaguerre *et al*. 2014.) *E*, errors between virtual inverse solution and real contact electrograms in human AF. The first labelled electrogram poorly matched the contact electrogram in timing and shape; the second matched well in timing but not in shape. Timing and shape both influence activation and phase maps. (From Schilling *et al*. 2000.) See text for further details.

Fibrillatory electrograms are typically analysed using rules developed for organized rhythms (Allessie *et al*. 2010; Houben *et al*. 2010; Lee *et al*. 2014) (Figs 2 and 3), yet resulting maps may be less accurate as rhythms progress from coherent (e.g. flutter) towards non‐spatially coherent fibrillation. Unipolar electrograms may be more accurate than bipolar, since adjacent (non‐coherent) activity is not subtracted (Steinhaus, 1989; Ndrepepa *et al*. 1995) although, conversely, they summate larger fields. Figure 5 *A* shows challenges in interpreting unipolar electrograms in AF, which have not been proven to represent local or far field activity by validation from optical maps. This may dramatically influence the results from AF maps.

## Optical mapping reveals localized sources for fibrillation (hierarchical model) across species including humans

Localized driving sources for fibrillation were postulated by Mines and Sir Thomas Lewis, then shown computationally (Krinksy, 1966). Spiral waves (rotors) have shown been to drive disordered activity in physical systems such as the Belousov–Zhabotinsky reaction (Winfree, 1972). Rotors have since been shown to be fibrillatory sources in ventricular tissue using optical mapping of fluorescent dyes (Davidenko *et al*. 1992; Witkowski *et al*. 1998), and have also been shown to drive AF in animal models (Mansour *et al*. 2001) and humans (Narayan *et al*. 2012 *d*; Hansen *et al*. 2015).

It is notable that optical mapping studies almost uniformly show that rotors sustain fibrillation, across model systems and species including humans, and sustain disorganized activity through a process termed fibrillatory conduction (Table 1). An optically mapped rotor is illustrated in Fig. 4. A rotor can be defined as a core or phase singularity (see below) from which spiral waves emanate at high speed to cause disorganization in surrounding tissue (Pandit & Jalife, 2013). This disorganization (fibrillatory conduction) may be due to factors such as steep APD restitution (Franz *et al*. 1988) conduction slowing (Kléber & Rudy, 2004) or tissue anisotropy (Valderrábano *et al*. 2001).

Optical imaging is well suited to map fibrillation because it simultaneously maps activation and recovery at high spatial resolution, with minimal cross‐talk and at high temporal resolution. Mathematical analyses are typically used to facilitate the quantification of organization within fibrillatory maps. Phase analysis developed by Gray *et al*. was first used to show organized reentrant sources in ventricular fibrillation (Gray *et al*. 1998) (Fig. 3 *B*). Phase represents the progression of tissue from activation onset through repolarization over time, which varies spatially and temporally during fibrillation. Spatial points around which phase exhibits a full rotation (i.e. –π to +π) have indeterminate phase and are termed singularities. Phase mapping is now validated across species for atrium and ventricle (Vaidya *et al*. 1999; Chen *et al*. 2000 *b*; Zaitsev *et al*. 2003; Noujaim *et al*. 2007).

This work has catalysed pivotal advances in understanding cardiac fibrillation. In recent decades, many studies have defined how spiral waves arise and drive fibrillation, attach to tissue heterogeneities such as fibrosis (Ikeda *et al*. 1997; Lim *et al*. 2006), and how organization breaks down at these edges into unstable fibrillatory activity (Pandit & Jalife, 2013). Rotors may also be detected by other techniques. For instance, periodicity indices such as dominant frequency after Fourier transform, have shown stable gradients in activation consistent with a rotor source (Zaitsev *et al*. 2000; Wu, 2002; Everett *et al*. 2004).

Rotors are traditionally considered distinct from functional reentry (leading circle) or anatomical reentry (Comtois *et al*. 2005), since rotors form due to extreme conduction slowing at the wave tip causing rotation around an ‘unexcited, yet eminently excitable’ core (Pandit & Jalife, 2013). However, spiral waves are well known to anchor to heterogeneities such as fibrosis and scar (Lin *et al*. 2008), and optical mapping of human AF shows stable endocardial micro‐reentry that sustain AF and precess near regions of micro‐fibrosis where localized ablation terminates AF (Hansen *et al*. 2015; Zhao *et al*. 2015). Mechanisms enabling the termination of AF by localized ablation are the subject of ongoing study (Rappel *et al*. 2015). Thus, the distinction between functional and structural reentry may continue to blur in human cardiac fibrillation (Zaman & Narayan, 2015).

In summary, the vast majority of studies in fibrillation that analysed action potentials – initially in animal ventricles, recently in human atria using optical maps – reveal localized sources that drive disorganization.

## Clinically identified mechanisms for human atrial fibrillation

Any proposed mechanistic model for human AF must explain clinical observations in patients including ablation. These include stable gradients of AF rate (Filgueiras‐Rama *et al*. 2012) and transient linking of activation (Gerstenfeld *et al*. 1992) within the fibrillating atria. Mechanisms must also explain how limited ablation may on occasion terminate persistent AF (Herweg *et al*. 2003), as increasingly reported by mechanism‐targeted mapping (Shivkumar *et al*. 2012; Narayan *et al*. 2012 *a*) yet, paradoxically, why extensive empirical ablation may not improve outcomes compared to more conservative approaches (Wynn *et al*. 2014; Verma *et al*. 2015).

Optical mapping has heightened the mechanistic debate in human AF. Optical mapping shows that AF in human right and left atria can be sustained by stable endocardial sources at micro‐fibrotic regions and terminated by local ablation (Fig. 3 *C*) (Hansen *et al*. 2015; Zhao *et al*. 2015). While these data agree with the wider optical mapping literature (Table 1) and some clinical studies showing localized sources, they disagree with the majority of clinical reports that support disorganized mechanisms without sources. The next few sections will attempt to reconcile these differences.

The vast majority of mapping studies in human AF over two decades have analysed unipolar (Allessie *et al*. 2010) or bipolar electrograms (Lee *et al*. 2014) rather than action potentials (MAP or optical data) (Table 1). In 24 patients with longstanding persistent AF and valvular disease at surgery, Allessie *et al*. analysed electrograms from atrial plaques and stated ‘… in over 4000 maps of persistent AF… failed to find any rotors or foci that could explain the persistence of AF’ (Allessie *et al*. 2010). Potential criticisms of this work are that no interventions were tested, the authors simultaneously mapped < 10% of each atrium (areas > 100 cm^2^ in Jadidi *et al*. (2013)) so could not exclude sources in remaining atrium, and the authors used electrogram analyses that are challenging (Figs 2, 3 and 5 *A*). More recently, this group reported rotational activity in AF (Fig. 5 *B*) (Lau *et al*. 2015), focusing on the limitations of analysing complex signals. Notably, Fig. 3 illustrates how challenging it may be to analyze signals in AF. Accordingly, some studies that failed to identify sources using wide‐area mapping are difficult to interpret showing, for example, very long (slow) cycle lengths of 250–500 ms (frequency 2–4 Hz) in patients with AF, suggesting analytic errors and the use of bipolar analyses (Shannon's entropy) on unipolar signals (Benharash *et al*. 2015). Another study of electrograms from epicardial plaques also found rotational circuits, albeit transient (Walters *et al*. 2015), yet with reproducible vectorial propagation in 62% of cases over prolonged periods that contradict the complexity and lack of rotational circuits in earlier electrogram studies (Allessie *et al*. 2010).

A novel approach for clinical mapping of AF termed focal impulse and rotor mapping (FIRM) has been designed to circumvent some limitations of traditional electrogram analyses. FIRM records endocardial signals simultaneously from widely sampled clinical electrodes, most practically a basket catheter. Computational analyses are then performed using monophasic APD and conduction restitution data applied to electrograms in human AF to physiologically filter far‐field signals (‘noise’), before phase analysis to identify activation patterns (Narayan *et al*. 2006, 2011 *b*, 2012 *b*; Krummen *et al*. 2012). Figure 5 *C* shows a stable endocardial AF rotor mapped at a typical FIRM case, that precesses in 1–2 cm^2^ areas, controls surrounding activity (Narayan *et al*. 2013), may be multiple (two to four) in each patient and can be eliminated by localized ablation with high long‐term AF elimination compared to conventional ablation (Narayan *et al*. 2014; Miller *et al*. 2014). One major limitation of FIRM is that although baskets are currently the most practical catheter inserted percutaneously to provide wide‐area contact recordings, basket design should be improved to increase resolution and contact. New designs (Lin *et al*. 2014) may improve upon earlier catheters. It is interesting to note that FIRM mapping results bear many similarities to human optical mapping studies of AF (Hansen *et al*. 2015; Zhao *et al*. 2015) (Table 1).

Figure 5 *D* shows an approach that records from a modified ECG (body surface vest) to mathematically infer unipolar epicardial electrograms, then applies phase to reveal AF drivers (Haissaguerre *et al*. 2014). These drivers are reported as ‘unstable’ – but cluster in reproducible regions for prolonged times where they can be successfully ablated (Haissaguerre *et al*. 2014). It is unclear why these results differ from other studies of human AF, i.e. whether wavefront instability in ECGI reflects variable epicardial activity from stable endocardial rotors (Fig. 3 *C*) (Hansen *et al*. 2015), or potentially technical factors from the inverse solution. Indeed, inverse solution virtual electrograms in AF may not reflect contact electrograms (Fig. 5 *E*) (correlation values as low as 0.27, average 0.87 in Schilling *et al*. (2000); Earley *et al*. 2006), and projecting epicardial circuits to the chest wall many centimetres away may magnify instability (Rodrigo *et al*. 2014).

Validation of mapping results in fibrillation is the subject of intense study. Traditionally, termination of an arrhythmia by ablation proves mechanism, but this is more difficult in AF where hundreds of lesions are often applied, and because clinically successful ablation may not acutely terminate AF (Elayi *et al*. 2010). Nevertheless, there are increasing reports of termination of persistent AF by localized ablation at a predicted location (Shivkumar *et al*. 2012; Narayan *et al*. 2012 *d*; Miller *et al*. 2014; Haissaguerre *et al*. 2014) that was previously rare. This observation supports localized sources for AF and is difficult to explain by non‐hierarchical ‘random’ mechanisms; it has stimulated studies on how localized ablation may terminate AF (Rappel *et al*. 2015) and why termination of AF by ablation may not predict long‐term success (Calkins *et al*. 2012).

In summary, whereas the action potential encodes information about activation and recovery, the electrogram focuses on activation. It is thus not surprising that different mapping approaches produce divergent results as the density of adjacent non‐coherent wavefronts increases, i.e. in fibrillation. Future studies that compare mapping approaches in clinical AF, particularly against optical maps, will help to further reconcile differences in observed mechanisms. A particularly important direction is to test whether ‘rules’ such as a ‘qS’ deflection indicating a focal source are accurate in AF when validated by MAP or optical data, and more importantly to improve algorithms for future mapping. Improved catheter designs may better represent local activation, and thus complement such algorithms.

## Mechanisms in human ventricular fibrillation

There are obvious challenges to mapping human VF, and studies have therefore focused on early VF (Wiggers stage I) prior to defibrillation, or patients on circulatory support. Nevertheless, within these limitations, fibrillatory mechanisms are well studied. At a tissue level, reentry in VF may depend on analogous processes to AF, including APD oscillations in animals with VF (Choi *et al*. 2001; Pastore *et al*. 2006) and patients at risk for VF (Koller *et al*. 2005; Narayan *et al*. 2008 *a*) and in VF (Nash *et al*. 2006 *a*). Dynamic conduction slowing has also been shown as a mechanism for the propensity to human VF in endocardial clinical (Narayan *et al*. 2008 *a*) and Langendorff‐perfused (Nanthakumar *et al*. 2007) studies.

**Figure 6:**
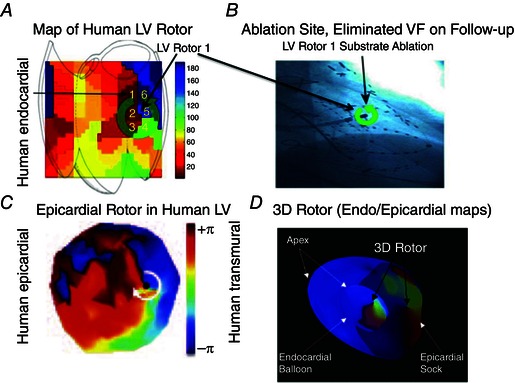
**Human VF rotors demonstrated using endocardial and epicardial mapping** *A*, FIRM mapping using basket electrograms shows human LV rotor 1. *B*, localized ablation at rotor rendered VF non‐inducible and eliminated VF on long‐term follow‐up. (From Krummen *et al*. 2015.) *C*, human VF epicardial rotor (white arrow) on phase map with complex fibrillatory breakdown. (From Nash *et al*. 2006 *b*.) *D*, transmural LV rotor (scroll wave) in *ex vivo* early human VF displayed using phase maps of endocardial and epicardial electrograms. (From Nair *et al*. 2011.)

In parallel with recent studies of human AF, studies of VF have attempted to identify hierarchical mechanisms (localized sources) (Fig. 6). Figure 6 *A* and *B* shows FIRM mapping of human VF prior to defibrillation, in which computational electrogram filtering using human APD and conduction restitution data revealed VF rotors in patients. Such rotors often arose in scar border zone (Hayase *et al*. 2013; Krummen *et al*. 2014, 2015), and proof of concept reports show that ablation of VF rotors (in sinus rhythm) was able to eliminate clinical VF on long‐term follow up (Hayase *et al*. 2013; Krummen *et al*. 2014, 2015). Using other mapping approaches, Fig. 6 *C* shows an epicardial rotor after 16.8 s of VF, and Fig. 6 *D* indicates a transmural rotor (scroll wave) in early VF using a combined phase map of endocardial and epicardial electrograms (Nash *et al*. 2006 *b*; Nair *et al*. 2011).

Structural factors may potentially distinguish the mechanisms for VF and AF, because complex ventricular architecture may anchor rotors, destabilize reentry or facilitate intramural mechanisms (Nash *et al*. 2006 *b*; Nair *et al*. 2011) as shown in simulations (Rogers, 2002). Transmural rotation of ventricular fibres from the epicardium to the endocardium (rotational anisotropy) (Thomas, 1957) may destabilize scroll waves to produce complex dynamics and wavebreak (Rappel, 2001). In patients at risk for sudden cardiac arrest, an ischaemic border zone may present thick–thin transitions and gap junction remodelling, which may also impact wavefront curvature and re‐entry (Macia *et al*. 2011; Ciaccio *et al*. 2014).

Thus, many studies of human VF support a hierarchical model driven by sources, yet these mechanisms are less well validated than for AF. First, it is less clear that VF rotors always act as sources, since VF is typically studied for short periods of time and long‐term follow‐up studies from VF rotor ablation, while promising, are presently anecdotal. Secondly, some ventricular studies used techniques that alter the substrate such as recording epicardial electrograms after sub‐endocardial ablation using Lugol's solution (Lee *et al*. 1996). Future directions in VF mapping include optical mapping studies to better define the structure–function relationship between organized and disorganized regions, and to validate diverse mapping approaches to VF.

## Conclusions and future directions

Knowledge in cardiac electrophysiology has progressed significantly in recent decades, yet remarkably, the fundamental mechanisms for human cardiac fibrillation are still debated. One important concept in this debate is that while fibrillation operates at the limits of dynamic activation and recovery, few human studies consider this physiology. Accordingly, mapping techniques that agree in simple rhythms may diverge dramatically in AF. Optical mapping typically shows localized rotors and sources driving fibrillation while activation mapping of electrograms typically simply shows disorganization.

These mechanistic differences have profound clinical implications. Therapy based upon the disorganized model of AF is suboptimal, and cannot easily explain abundant observations of AF modulation by localized intervention or the failure of extensive empiric ablation to improve outcomes. Conversely, novel clinical mapping approaches, including FIRM that computationally combines repolarization with activation data, recapitulate many features of fibrillatory sources found in human optical maps. Therapy based upon novel mechanistic approaches to mapping have shown early promise and randomized trials are underway.

Future directions include, ultimately, the development of clinically useable optical imaging, which circumvents dye‐related toxicity in the beating heart at wavelengths not absorbed by blood. In the interim, optical mapping of human AF *ex vivo* should be used to validate and compare current mapping modalities, to develop accurate ‘rules’ or algorithms to separate local from distant information for the different recording fields of unipolar or bipolar electrograms. Improved catheter designs may better represent local activation, and complement such algorithms. These approaches may ultimately lead to computational mapping that digitally approximates optical maps.

Recent literature in cardiac fibrillation is replete with studies that are descriptive without mechanistic interventions. For the essential goal of advancing clinical outcomes, it is critical that purely descriptive studies are replaced by studies in which ablation and other interventions are used to establish mechanistic targets for diagnosis and for novel therapies which may include ablation, pacing, genetic or regenerative therapy.

## Additional information

### Competing interests

S.M.N. reports being co‐inventor on intellectual property owned by the University of California and licensed to Topera Medical, Inc. He has held equity in Topera. S.M.N. also reports having received consulting fees from the American College of Cardiology, Uptodate and Janssen Pharmaceuticals. J.A.B.Z. reports no competing interests.

### Author contributions

Both authors contributed substantially to the conceptualization, drafting, revision and assembly of the manuscript. Both authors approved the final version of the manuscript, both persons designated as authors qualify for authorship, and all those who qualify for authorship are listed.

### Funding

This work was supported in part by grants from the National Institutes of Health (HL70529, HL83359, HL103800) to S.M.N., and a British Heart Foundation Fellowship (FS/14/46/30907) and Fulbright Award to J.Z.
